# Reduced temporal muscle thickness predicts shorter survival in patients undergoing chronic subdural haematoma drainage

**DOI:** 10.1002/jcsm.13489

**Published:** 2024-05-08

**Authors:** Tommi K. Korhonen, Otso Arponen, Moritz Steinruecke, Ilaria Pecorella, Harry Mee, Stefan Yordanov, Edoardo Viaroli, Mathew R. Guilfoyle, Angelos Kolias, Ivan Timofeev, Peter Hutchinson, Adel Helmy

**Affiliations:** ^1^ Division of Neurosurgery, Department of Clinical Neurosciences Cambridge University Hospitals NHS Foundation Trust & University of Cambridge Cambridge UK; ^2^ Department of Neurosurgery, Neurocenter OYS Oulu University Hospital Oulu Finland; ^3^ Department of Neurosurgery, Research Unit of Clinical Neurosciences University of Oulu Oulu Finland; ^4^ Department of Radiology University of Cambridge Cambridge UK; ^5^ Faculty of Medicine and Health Sciences Tampere University Tampere Finland; ^6^ Department of Radiology Tampere University Hospital Tampere Finland

**Keywords:** Body composition, Chronic subdural haematoma, Computed tomography, Frailty, Sarcopenia

## Abstract

**Background:**

Chronic subdural haematoma (CSDH) drainage is a common neurosurgical procedure. CSDHs cause excess mortality, which is exacerbated by frailty. Sarcopenia contributes to frailty – its key component, low muscle mass, can be assessed using cross‐sectional imaging. We aimed to examine the prognostic role of temporal muscle thickness (TMT) measured from preoperative computed tomography head scans among patients undergoing surgical CSDH drainage.

**Methods:**

We retrospectively identified all patients who underwent CSDH drainage within 1 year of February 2019. We measured their mean TMT from preoperative computed tomography scans, tested the reliability of these measurements, and evaluated their prognostic value for postoperative survival.

**Results:**

One hundred and eighty‐eight (122, 65% males) patients (median age 78 years, IQR 70–85 years) were included. Thirty‐four (18%) patients died within 2 years, and 51 (27%) died at a median follow‐up of 39 months (IQR 34–42 months). Intra‐ and inter‐observer reliability of TMT measurements was good‐to‐excellent (ICC 0.85–0.97, *P* < 0.05). TMT decreased with age (Pearson's *r* = −0.38, *P* < 0.001). Females had lower TMT than males (*P* < 0.001). The optimal TMT cut‐off values for predicting two‐year survival were 4.475 mm for males and 3.125 mm for females. TMT below these cut‐offs was associated with shorter survival in both univariate (HR 3.24, 95% CI 1.85–5.67) and multivariate (HR 1.86, 95% CI 1.02–3.36) analyses adjusted for age, ASA grade and bleed size. The effect of TMT on mortality was not mediated by age.

**Conclusions:**

In patients with CSDH, TMT measurements from preoperative imaging were reliable and contained prognostic information supplemental to previously known predictors of poor outcomes.

## Introduction

Surgical drainage of a chronic subdural haematoma (CSDH) is one of the most common neurosurgical operations.^1^ The incidence of CSDH is increasing as populations age, contributing to excess morbidity and mortality.^2^ Patients with CSDH have a mortality rate of 14–32% in the first postoperative year^3^, ^4^ and excess mortality is sustained up to 20 years after diagnosis.^3^ In fact, CSDH has been regarded as a ‘sentinel event’ which manifests underlying medical comorbidity.^4^ Complementary to improving treatment strategies,^5^, ^6^, ^7^, ^8^ identifying particularly vulnerable CSDH patients would allow interventions to be targeted toward those most at risk. Adverse outcomes among patients with neurosurgical conditions, such as CSDH, traumatic brain injury and central nervous system tumours, are mediated by sarcopenia, frailty, and malnutrition.^3^, ^9^, ^10^, ^11^, ^12^, ^13^, ^14^, ^15^

Low muscle mass is a characteristic of various geriatric syndromes, such as sarcopenia, frailty and malnutrition.^16^ Temporal muscle thickness (TMT), measured on diagnostic computed tomography (CT) head scans, correlates with muscle measurements performed at the level of the third lumbar vertebra,^17^, ^18^, ^19^ which is a widely studied marker of adverse outcomes and survival in oncological and surgical patients.^20^, ^21^, ^22^ There is a paucity of studies evaluating the association between low TMT and survival following CSDH drainge. Previously, Dubinski et al. found an association between reduced TMT and poor performance status following CSDH surgery in a univariate analysis.^23^ Here, we aimed to evaluate the prognostic value of TMT for overall survival following CSDH surgery, and to establish sex‐specific cut‐off values for TMT to assess two‐year mortality. To study the underlying mechanisms between muscle mass and mortality, we also examined the relationship between TMT and age, sex, and well‐established radiological characteristics, namely, CSDH volume and the resulting midline shift.

## Materials

### Study cohort

**Figure 1:**
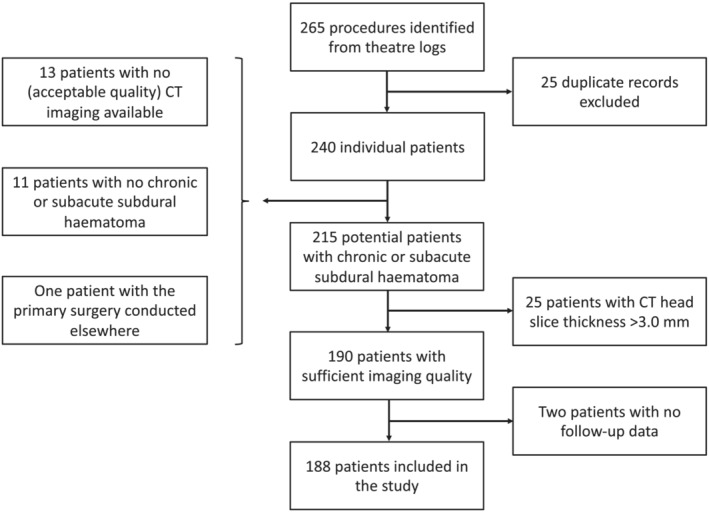
Study flowchart.

This was a retrospective cohort study comprised of consecutive patients who underwent surgical drainage of CSDH at Cambridge University Hospitals (CUH) between 1 February 2019 and 28 February 2020 (*n* = 188, Figure 1). This study was part of a clinical audit aiming to evaluate clinical vulnerabilities in patients with CSDH admitted to CUH. We included consecutive patients who underwent their first surgery for a CSDH predominantly consisting of radiologically hypo‐ or isodense fluid in a preoperative CT head scan and had surgical findings consistent with CSDH. Patients were excluded if the CT scans were contrast‐enhanced, had significant imaging artefact, the CT slice thickness was >3.0 mm, or mortality data was unavailable.

### Clinical parameters

Clinical and laboratory data were collected from electronic patient records. Clinical data included demographics, the type of surgery, and the preoperative American Society of Anesthesiologists Physical Classification Scale (ASA), assessed by the anaesthetist. Mortality data was obtained from the National Health Service Spine portal, a centralised patient registry with national coverage in the United Kingdom. Mortality data was not available for two (1%) patients, which led to their exclusion (Figure 1). Follow‐up time was calculated as the time between surgery and death or 31 December 2022, whichever occurred sooner.

### Surgery

CSDHs were evacuated through cranial burr holes or a craniotomy. In the former, a 2–3 cm scalp incision was made over the maximum extent of the intracranial bleed, a 14 mm burr hole was made to the skull, the dura opened, and the subdural space thoroughly irrigated with room‐temperature saline while the patient was under general anaesthesia. A drain was inserted subdurally and tunnelled through the scalp, followed by layered closure of the scalp. A larger opening of the skull, craniotomy, was conducted if the bleed appeared membranous or otherwise difficult to evacuate via a burr hole, or if intraoperative findings necessitated extending the skull opening. The craniotomy procedure was otherwise similar to burr hole CSDH evacuation but conducted through a longer incision and a 3–5 cm circular skull opening to achieve better exposure and surgical control of the CSDH. A subdural drain was then placed and the craniotomy bone flap was reaffixed to the skull using a plating kit or surgical clamps, followed by a layered closure of the scalp. The surgical drain was removed bedside once no more subdural fluid was expelled, usually after 12–48 hours, and a suture was placed to close the drain passage.

### Radiological measurements

**Figure 2:**
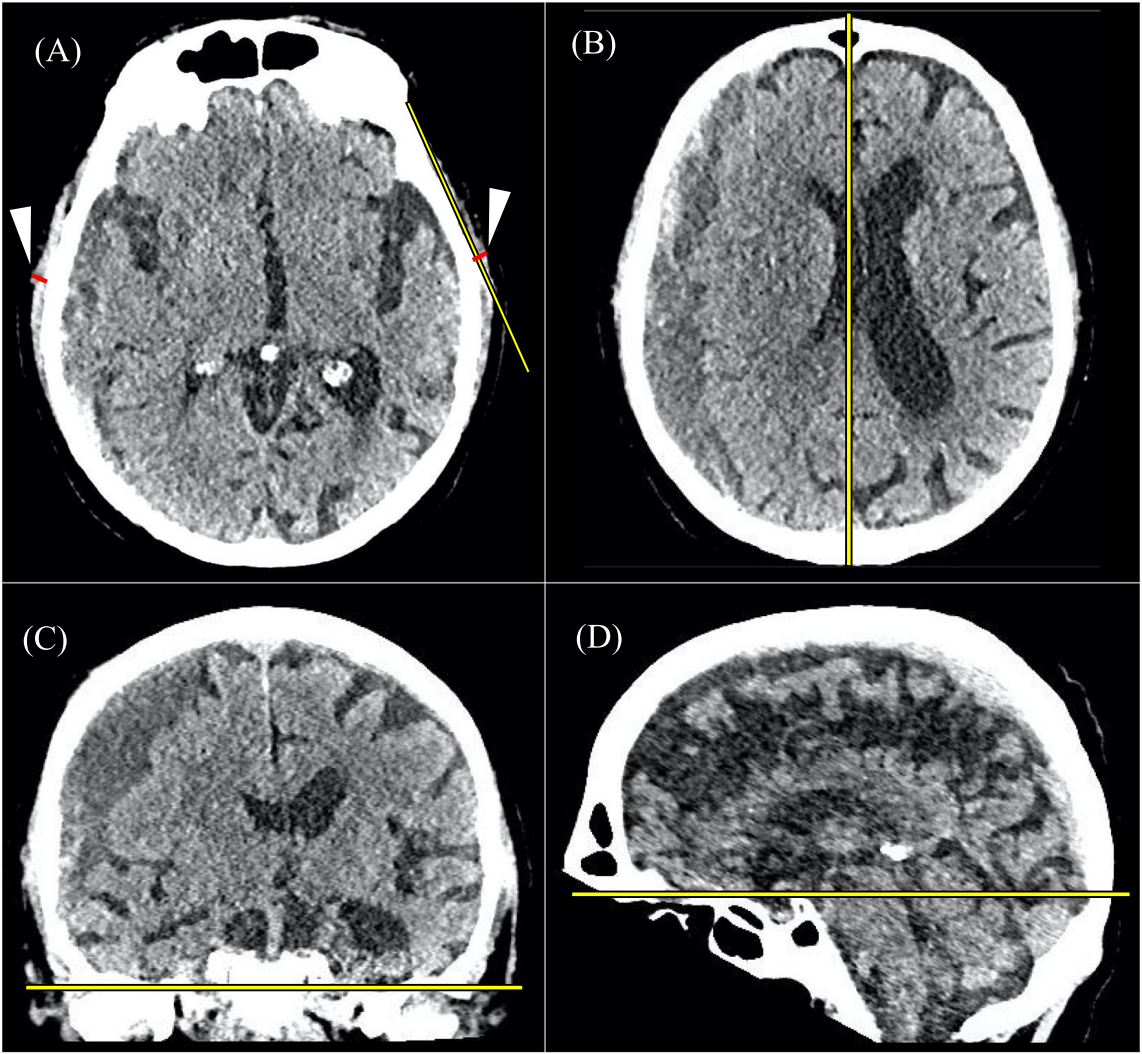
(A) The semi‐axial plane used for the temporal muscle measurements. The yellow line connects the most medial part of the bony temporal fossa and the most lateral part of the temporoparietal bone without intersecting the cranium. The temporal muscle measurements (red lines, white arrowheads) were made perpendicular to this line on the thickest part of the temporal muscle. (B–D) The axial, coronal and sagittal localisation planes used to align the scan to produce the sem‐iaxial plane A. The yellow lines are reference lines connecting the insertions of the falx cerebri (B), parallel to the middle fossa floor (C), and the anterior skull base (D).

A neurosurgeon [T. K. K. (*N* = 96)] and a radiologist [O. A. (*N* = 92)] independently measured TMT, midline shift and bleed size on the patients' most recent CT head scan prior to CSDH surgery with the McKesson Radiology Clinical Reference Viewer (McKesson Technology Solutions, GA, USA). Using the three multiplanar reconstruction dimensions, the measurement plane was oriented parallel to the anterior skull base (semi‐axial plane), parallel to the falx cerebri (sagittal plane), and tangential to the floor of the middle cranial fossae (coronal plane) (Figure 2). The semi‐axial plane from the brain sequence was windowed to linear width 80 and length 40 and used for all radiological measurements. After conducting the measurements in 10 patients, a meeting was held to confirm initial agreement between the raters. Discrepancies in the measurement process were resolved by the senior author (A.H.).

#### Temporal muscle thickness

TMT was measured on the first axial slice above the bony orbit. Suitable semi‐axial measurement planes were identified individually for the left and right sides of the head. A line connecting the most medial part of the temporal fossa and the most lateral part of the temporoparietal bone was drawn without intersecting the cranium, and the TMT was recorded as the thickest part of the temporal muscle perpendicular to this line (Figure 2). Where discernible, fat, fascia and vascular structures were not included in the measurement. The average of the left and right TMT measurements was used in the analyses.

#### Other radiological measurements

Midline shift was measured as the longest perpendicular distance of the septum pellucidum from a sagittal line connecting the anterior and posterior bony insertions of the falx cerebri on the level of the lateral ventricles in the semi‐axial plane, as defined above. The bleed volume was measured using the ABC/2 method.^24^ For bilaterally operated patients, the CSDH volume on each operated hemisphere was summed to calculate the total operated bleed volume.

#### Reliability and repeatability

To assess the reliability of the measurements, the two raters re‐measured TMT from 48 head CT head scans randomly chosen from the study dataset.

### Statistical analyses

Patient demographics were presented as absolute values and percentages. Continuous variables were presented as means with standard deviations (SDs) and medians with interquartile ranges (IQRs). Variable distributions were compared using the Kruskal–Wallis and Mann–Whitney *U* tests. Linear correlation between age and TMT was assessed using Pearson's correlation coefficient.

The intra‐ and inter‐observer reliability analyses of TMT measurements were assessed with the intraclass correlation coefficient (ICC). The ICCs were reported with the associated 95% confidence intervals (CIs) and interpreted as follows: ICCs <0.50 indicated poor, 0.50–0.75 moderate, 0.75–0.90 good, and >0.90 excellent reliability. Cohen's kappa values for TMT cut‐off reliability were determined and interpreted as follows: <0 indicated poor, 0.0–0.20 slight, 0.21–0.40 fair, 0.41–0.60 moderate, 0.61–0.80 substantial and 0.81–1.0 almost perfect agreement.^25^

We determined sex‐specific cut‐off values of TMT for predicting two‐year mortality using receiver operator characteristic (ROC) curves by maximising Youden's index. Binary logistic regression odds ratios (ORs) and CIs were used to examine associations between TMT and patient characteristics. The association between TMT and survival was examined using Cox regression modelling, reported with hazard ratios (HRs) and 95% CIs. We analysed the interaction term between age and TMT and included it in the multivariate survival models due to the well‐described correlation of age and muscle mass.^26^

All the *P*‐values were obtained from two‐sided tests, and they were considered statistically significant when <0.05. Data were analysed using IBM SPSS (Version 27.0.0.1, Armonk, USA).

## Results

### Cohort description

One hundred and eighty‐eight patients (122 (65%) males and 66 (35%) females) were included in the study. The median age of the patients was 78 years (IQR 70–85), and the median follow‐up time was 3 years and 2 months (IQR 33–42 months). Thirty‐four (18%) patients died within 2 years of surgery, and 51 (27%) had died by the end of follow‐up. Twenty‐two (12%) patients had at least one re‐operation: 18 (10%) due to haematoma recurrence and four (2%) due to infection. The detailed baseline characteristics stratified by two‐year and overall mortality are presented in Table 1.

**Table 1:** Baseline characteristics of 188 patients who underwent surgical CSDH drainage.

Characteristic	Two‐year mortality		Overall mortality		Total (*n* = 188)	HR (95% CI)
	Alive (*n* = 154)	Deceased (*n* = 34)	Alive (*n* = 137)	Deceased (*n* = 51)		
Age (years), mean (SD)	**73.5 (13.6)**	**85.3 (6.9)**	**72.6 (13.6)**	**51 (9.2)**	**75.6 (13.5)**	**1.10 (1.06–1.14)**
Male, *n* (%)	96 (62.3)	26 (76.4)	84 (61.3)	38 (74.5)	122 (64.9)	1.69 (0.90–3.17)
ASA grade, mean (SD)^a^	**2.7 (0.7)**	**3.4 (0.7)**	**2.7 (0.8)**	**3.2 (0.7)**	**2.9 (0.8)**	**2.09 (1.49–2.93)**
Body mass index (kg/m^2^), mean (SD)^a^	25.6 (6.9)	23.4 (4.2)	25.6 (6.2)	24.4 (7.4)	25.2 (6.5)	0.97 (0.91–1.03)
Bilateral surgery, *n* (%)	14 (9.1)	8 (23.5)	13 (9.5)	9 (17.6)	166 (88.3)	1.84 (0.90–3.79)
Burr hole surgery, *n* (%)^b^	121 (78.6)	29 (85.3)	107 (78.1)	43 (84.3)	150 (79.8)	1.39 (0.65–2.95)
TMT (mm), mean (SD)	**4.9 (1.7)**	**3.8 (1.4)**	**4.9 (1.7)**	**4.0 (1.7)**	**4.7 (1.7)**	**0.73 (0.61–0.87)**
TMT ≤ sex‐specific cut‐off, *n* (%)^c^	**44 (28.6)**	**22 (64.7)**	**36 (26.3)**	**30 (58.8)**	**66 (35.1)**	**3.24 (1.85–5.67)**
Midline shift (mm), mean (SD)	8.9 (4.4)	8.6 (5.2)	9.1 (4.4)	8.1 (4.7)	8.8 (4.5)	0.96 (0.90–1.02)
CSDH volume (cm^3^), mean (SD)	**134.4 (56.0)**	**187.1 (90.5)**	**132.1 (52.9)**	**175.8 (86.6)**	**144.0 (66.5)**	**1.01 (1.00–1.01)**

Statistically significant HRs of univariate Cox overall mortality analyses are bolded.

ASA, American Society of Anesthesiologists Physical Status Classification System; CI, confidence interval; CSDH, chronic subdural haematoma; HR, hazard ratio; SD, standard deviation; TMT, temporal muscle thickness.

^a^ The ASA grade was available for 183 (97%; 134 alive, 49 deceased), body mass index for 156 (82%; 125 alive, 31 deceased) patients.

^b^ Compared with minicraniotomy (*n* = 38).

^c^ 4.475 mm and 3.125 mm for male and female patients, respectively.

### Temporal muscle measurements and clinicoradiological variables

**Figure 3:**
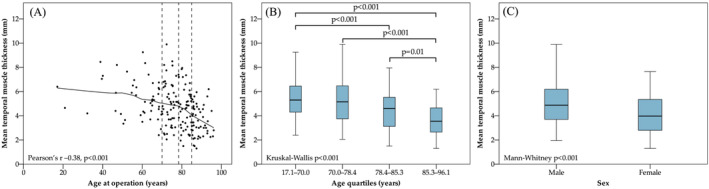
(A) Age‐related distribution of mean temporal muscle thickness among 188 patients with surgically managed chronic subdural haematoma. A locally weighted scatterplot smoothing curve (unbroken line) is shown. The dashed lines represent the upper limits of the first, second, and third age quartiles from left to right (at 70.0, 78.4, and 85.3 years, respectively). Temporal muscle thickness was inversely correlated with age at operation. (B) Boxplot of mean temporal muscle thicknesses of each age quartile. Only between‐group comparisons with *P* < 0.05 are shown. (C) Boxplot showing that male patients had thicker temporal muscles than female patients.

The intra‐ and inter‐observer reliability of the TMT measurements was good‐to‐excellent (ICCs 0.85–0.94 and 0.89–0.97, respectively; Table S1). TMT was inversely correlated with age at operation, and male patients had higher TMT than female patients (Figure 3).

**Figure 4:**
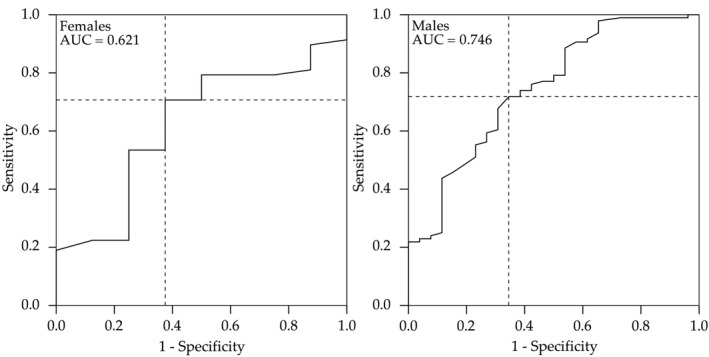
The receiver operating characteristic (ROC) curves for temporal muscle thickness (TMT) used to predict 2‐year mortality. The diagonal lines are reference lines demonstrating equal sensitivity and specificity, and the intersection of the dashed lines represents the highest Youden's index calculated from the ROC curve. The resulting TMT cut‐off values were 4.475 mm for males and 3.125 mm for females. AUC, area under the curve.

The optimal sex‐specific TMT cut‐off values for predicting two‐year mortality were calculated by maximising Youden's index (Figure 4). The cut‐off values were 4.475 mm for male and 3.125 mm for female patients. Using these cut‐offs, raters 1 and 2 classified 34/96 (35%) and 32/92 (35%) patients as having low muscle mass, respectively. The intra‐observer and inter‐observer agreements using this cut‐off were 0.64–0.86 and 0.55–0.76 as shown in Table S1.

Patients with TMT below the sex‐specific cut‐off values had higher two‐year and overall mortality than those with TMT above the cut‐off (33% vs. 10% and 45% vs. 17%, respectively, *P* < 0.001 for both comparisons). Patients with TMT below the sex‐specific cut‐off values were also older at the time of surgery and had higher CSDH volumes, whereas ASA grade, preoperative midline shift, type of surgery or body mass index were not associated with having a TMT above or below the cut‐off values (Table 2).

**Table 2:** Univariate binary logistic regression analysis of factors associated with TMT below or equal to the sex‐specific cut‐off values of 4.475 mm and 3.125 mm for male and female patients with CSDH, respectively

Characteristic	TMT ≤ cut‐off (*n* = 66)	TMT > cut‐off (*n* = 122)	OR (95% CI)
Age (years), mean (SD)	**81.5 (9.7)**	**72.4 (14.1)**	**1.07 (1.04–1.11)**
Male, *n* (%)	44 (67)	78 (64)	1.13 (0.60–2.12)
ASA grade, mean (SD)^a^	2.9 (0.8)	2.8 (0.7)	1.18 (0.80–1.74)
Body mass index (kg/m^2^), mean (SD)^a^	24.1 (6.7)	25.8 (6.4)	0.95 (0.89–1.02)
Bilateral surgery, *n* (%)	11 (17)	11 (9)	2.02 (0.82–4.94)
Burr hole surgery, *n* (%)^b^	57 (86)	93 (76)	1.98 (0.87–4.47)
Midline shift (mm), mean (SD)	8.9 (4.4)	8.8 (4.6)	1.00 (0.94–1.07)
CSDH volume (cm^3^), mean (SD)	**160.5 (63.3)**	**135.0 (66.7)**	**1.01 (1.00–1.01)**

Statistically significant comparisons are bolded.

ASA, American Society of Anesthesiologists Physical Status Classification System; CI, confidence interval; CSDH, chronic subdural haematoma; OR, odds ratio; SD, standard deviation; TMT, temporal muscle thickness.

^a^ ASA grade was available for 183 (97%; 134 alive, 49 deceased), body mass index for 156 (83%; 125 alive, 31 deceased) patients.

^b^ Compared with craniotomy (*n* = 38).

### Survival

**Figure 5:**
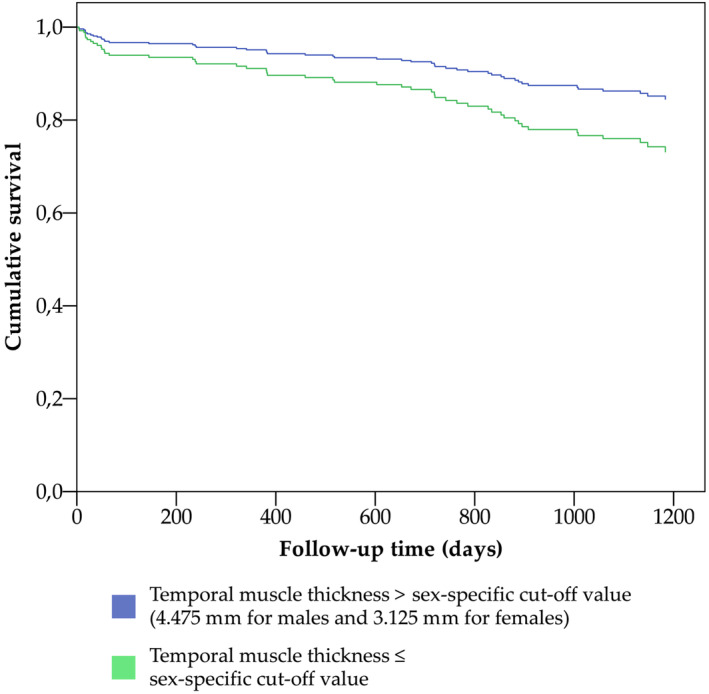
Cox regression survival curves for overall survival following chronic subdural haematoma surgery stratified by the sex‐specific cut‐off values of temporal muscle thickness. Variables included in the model were TMT according to the sex‐specific cut‐off values, age at operation, American Society Anesthesiologists Physical Classification Scale score and haematoma volume (Table 3, Model 2).

Reduced TMT, older age at the time of surgery, high ASA grade, and high CSDH volume were associated with shorter survival in univariate Cox regression analyses, whereas sex, body mass index, preoperative midline shift, bilaterality and the type of surgery were not (Table 1). In multivariate Cox regression analyses, TMT was associated with shorter survival when adjusted for age alone (Table 3, Model 1), and for age, ASA grade and CSDH volume (Table 3, Model 2; Figure 5). No statistically significant interactions between age and TMT were found when Model 1 (HR 1.02, 95% CI 0.95–1.10) or Model 2 (HR 1.03, 95% CI 0.95–1.11) were adjusted for the interaction term.

**Table 3:** Multivariate cox regression analysis of overall survival of patients who underwent surgical CSDH drainage.

Characteristic	HR (95% CI)
Model 1	
TMT ≤ sex‐specific cut‐off	**2.02 (1.13–3.64)**
Age at operation (years)	**1.09 (1.05–1.13)**
Model 2	
TMT ≤ sex‐specific cut‐off	**1.86 (1.02–3.36)**
Age at operation (years)	**1.08 (1.04–1.12)**
ASA grade^a^	**1.77 (1.25–2.50)**
CSDH volume (cm^3^)	**1.00 (1.00–1.01)**

Statistically significant HRs of univariate Cox overall mortality analyses are bolded.

ASA, American Society of Anesthesiologists Physical Status Classification System; CI, confidence interval; CSDH, chronic subdural haematoma; HR, hazard ratio; TMT, temporal muscle thickness.

^a^ ASA grade was available for 183 (97%; 134 alive, 49 deceased) patients.

## Discussion

We showed that reduced TMT on preoperative CT scans is associated with two‐year and overall mortality following CSDH surgery and provides information supplemental to known predictors of outcome. The overall and two‐year mortality rates of patients with TMT below our sex‐specific cut‐off values was significantly higher than that of those with TMT above the cut‐off value (33% vs. 10% and 45% vs. 17%, respectively). In addition, we found that the intra‐ and interrater reliability of TMT measurements was good‐to‐excellent, and that TMT was associated with sex and age.

### Temporal muscle thickness in neurological disorders

Imaging‐detected low muscle mass measured at the lumbar level, especially at the level of the third lumbar vertebra, has been established as a marker of poor outcomes in oncological and surgical patients.^20^ Recently, TMT measurements have been shown to correlate with L3‐level measurements.^17^, ^18^, ^19^ The only prior work on TMT in patients with CSDH by Dubinski et al. did not report mortality data. However, they reported that TMT categorised as low (1‐5 mm) or normal (>5 mm) was associated with a higher degree of disability at discharge and at 3 months. Notably, this association did not remain significant in their multivariate model.^23^

We sought to assess medium‐term mortality and establish sex‐specific cut‐off values for low and normal TMT for patients with CSDH instead of focusing on short‐term outcomes. We found that patients with low TMT (males/females: <4.475/<3.125 mm) had three‐fold higher two‐year and overall mortality rates compared to those with normal TMT. Although this is the first study to suggest a prognostic role for TMT measurements in CSDH, previous observations have associated low TMT with poor prognosis in glioblastoma multiforme,^27^ central nervous system lymphoma,^18^ stroke,^28^ and following endovascular thrombectomy,^29^ supporting our results.

### Clinical significance

Our findings and the existing literature on the imaging‐detected loss of muscle mass suggest that TMT may be a surrogate for overall clinical and nutritional status.^30^, ^31^ Radiologically‐assessed muscle mass was a predictor of mortality following CSDH surgery independent of chronological age. Indeed, muscle mass appears to reflect biological rather than chronological age, possibly explaining its prognostic effect across all age groups. This builds on the body of evidence associating reduced muscle mass with sarcopenia, frailty, and malnutrition,^16^ all of which are geriatric syndromes and contribute to poor patient outcomes.^32^, ^33^, ^34^ We believe that the association between low muscle mass and reduced survival may be useful in resource allocation and individually tailoring treatments.

#### Resource allocation

The recognition of frailty is clinically important, as it predicts poor clinical outcomes and lengthened hospital stays in surgical patients.^35^, ^36^ Our findings suggest that CT head scans could be used for opportunistic screening of patients with CSDH. Low TMT on preoperative CT scans could mandate a clinical assessment for causes of reduced muscle mass, based on which intensified geriatric care could be offered to support postoperative recovery and reduce falls risk.^37^, ^38^ Such a protocol could enable cost‐effective resource allocation, potentially reducing CSDH recurrence and improving clinical outcomes. Nevertheless, the clinical benefit from such screening and directed therapy protocols aimed at increasing muscle mass remains to be established.

#### Individually tailored treatment

The decision to operate is made if the patient is expected to have sufficient recovery capacity to benefit from the procedure. This assessment is often conducted in a remote consultation based on teleradiological findings and indirect clinical information from a referring institution, with shortcomings in the description of the patient's baseline condition potentially hampering operative decision‐making.^39^ Our results suggest that the assessment of TMT may be useful for the preoperative prognostication of patients with CSDH and could be considered alongside established radiological parameters, such as midline shift and bleed size, and other clinical information.

### Future directions

Increasingly diverse management options for CSDH may require more accurate assessment of patients' suitability for different treatment modalities in the future. Recent trials have refined contemporary surgical techniques,^5^, ^7^, ^8^ but further personalisation of CSDH management is anticipated. Pending confirmation from ongoing trials, middle meningeal artery embolisation is expected to become a novel endovascular CSDH treatment option, obviating the need for surgical interventions and associated recovery,^6^ which may be particularly beneficial for frail patients. In this context, an objective measure of vulnerability, such as TMT, could help in clinical decision‐making between treatment options.

Future research should assess the value of TMT in traumatic brain injuries other than CSDH. Accurate prognostication may be even more important in more severe types of brain injury, which require more invasive surgery and therefore more demanding rehabilitation than CSDH. While age is commonly used as an exclusion criterion for invasive surgery,^40^ methods to identify older patients who may still benefit and recover from treatment are needed.

Although the manual measurement of TMT was feasible, artificial intelligence‐based methods could be applied to aid in the automated screening of reduced muscle mass in the future. Although no obvious technical barriers exist, no such algorithms for the measurement of TMT from clinical CT scans have been published.

## Limitations

Prospective studies are needed to validate our results and evaluate the underlying relationships between reduced TMT and survival. Since our study did not include conservatively managed patients, it remains to be established whether TMT could aid in decision‐making around operative and conservative treatments. Given the associations between TMT, age and sex, normative cut‐off values standardised by these parameters would be useful in clinical practice and future research. Finally, the clinical benefit of TMT‐based screening of muscle mass and targeted supportive therapy protocols aimed at increasing muscle mass remains to be established.

## Conclusions

Among patients undergoing surgery for CSDH, TMT measurements from preoperative clinical imaging were reliable and contained prognostic information supplemental to known predictors of poor outcomes.

## Conflict of interest

The authors have no relevant conflicts of interest to declare.

## Funding

The study received no funding. Authors' support: TKK–The Finnish Medical Foundation (grant number 4268), The Finnish Cultural Foundation (grant number 00221201), Orion Research Foundation sr; OA–The Osk. Huttunen Foundation, Sigrid Jusélius Foundation, Relander Foundation, Finnish Medical Foundation, Cancer Foundation Finland, and Orion Research Foundation; PJ–National Institute for Health Research (Professorship, Biomedical Research Centre, Brain Injury MedTech Co‐operative, Senior Investigator Award and the Royal College of Surgeons of England); AH–the NIHR Biomedical Research Centre, the NIHR Brain Injury MedTech Co‐operative, and Royal College of Surgeons of England. The funders had no role in the design of this study, its execution, analyses, interpretation of the data, or the decision to publish the results.

## Supporting information


**Table S1.** Intra‐ and inter‐observer reliability analysis of the mean TMT measurements assessed using the ICC, and intra‐ and inter‐observer reliability analysis of the TMT status assessed (over/under the sex‐specific cut‐off values) using the Cohen's kappa coefficient (*n* = 48).

## Data Availability

All the relevant data is published in the article. Original analyses without patient‐level data can be requested by submitting a request to the Division of Neurosurgery at the University of Cambridge. All appropriate information governance protocols will be followed.
