# Supplementary material for: Reduced temporal muscle thickness predicts shorter survival in patients undergoing chronic subdural haematoma drainage
Source: J Cachexia Sarcopenia Muscle. 2024 May 8;15(4):1441–50. doi: 10.1002/jcsm.13489 (PMC11294050; doi:10.1002/jcsm.13489)
Supplement: Supplementary file 1 — Table S1. Intra‐ and inter‐observer reliability analysis of the mean TMT measurements assessed using the ICC, and intra‐ and inter‐observer reliability analysis of the TMT status assessed (over/under the sex‐specific cut‐off values) using the Cohen's kappa coefficient (n = 48). [file JCSM-15-1441-s001.docx]

| **Supplementary Table 1.** Intra- and inter-observer reliability analysis of the mean TMT measurements assessed using the ICC, and intra- and inter-observer reliability analysis of the TMT status assessed (over/under the sex-specific cut-off values) using the Cohen’s kappa coefficient (n=48). | | |
| --- | --- | --- |
| **Comparison** | **ICC (95% CI)** | **Kappa value** |
| **Intra-observer** |  |  |
| Rater 1.1 v. Rater 1.2 | 0.97 (0.93–0.98) | 0.86 |
| Rater 2.1 v. Rater 2.2 | 0.89 (0.81–0.94) | 0.64 |
| **Inter-observer** |  |  |
| Rater 1.1 v. Rater 2.1 | 0.92 (0.85–0.95) | 0.72 |
| Rater 1.2 v. Rater 2.2 | 0.87 (0.76–0.93) | 0.68 |
| Rater 1.1 v. Rater 2.2 | 0.85 (0.75–0.91) | 0.55 |
| Rater 1.2 v. Rater 2.1 | 0.94 (0.89–0.96) | 0.76 |
| TMT = temporal muscle thickness, ICC = intraclass correlation coefficient, CI = confidence interval. Rater 1.1 = the first rating round of Rater 1, Rater 1.2 = the second rating round of Rater 1, Rater 2.1 = the first rating round of Rater 2, Rater 2.2 = the second rating round of Rater 2. *In respective order. | | |
